# The magnitude of failed induction and associated factors among women admitted to Adama hospital medical college: A cross-sectional study

**DOI:** 10.1371/journal.pone.0262256

**Published:** 2022-01-27

**Authors:** Bikila Tefera Debelo, Reta Nemomsa Obsi, Worku Dugassa, Shumi Negasa

**Affiliations:** 1 Department of Midwifery, College of Medicine and Health Sciences, Ambo University, Ambo, Ethiopia; 2 Department of OBGYN, Adama Hospital Medical College, Adama, Ethiopia; 3 Department of Public Health, Adama Hospital Medical College, Adama, Ethiopia; Universita Politecnica delle Marche, ITALY

## Abstract

**Introduction:**

Induction of labor is a medical iatrogenic stimulation of uterine contraction before the spontaneous onset of labor to achieve vaginal delivery. It is an increasingly being done obstetric procedure throughout the world and associated with poorer outcomes when compared with spontaneous labor. The published evidence is limited in Ethiopia including the study area. Therefore, this study was aimed at assessing the magnitude of failed induction and associated factors among pregnant women who were admitted to the labor ward of Adama hospital medical college.

**Methods:**

Institution-based cross-sectional study was conducted among 293 women who were eligible for induction using systematic random sampling. The data were collected from 1st January to 30th April 2020 by face-to-face interview using a structured questionnaire and extraction from a maternal chart. Then data was entered into Epi-data version 4.6 and analyzed using Statistical Product and Service Solution version 23. Descriptive statistics were performed to describe the study population. Logistic regression (bivariate and multivariable) analysis was conducted to identify associated factors. The association was expressed in odds ratio with 95% confidence interval and P-value <0.05 was used as cut-off points to declare significance in the final model.

**Results:**

This study showed that the prevalence of failed induction was 20.5% (95% CI: (15.7–25.3%)). The odds of failed induction in unfavorable bishop score were 4.05 higher than the odds in favorable bishop [AOR = 4.05 95%CI (1.19–13.77)]. The odds of failed induction in an intact membrane were 2.05 higher than the ruptured membrane. [AOR = 2.05, 95%CI (1.06–3.98)]. The odds of failed induction in primigravida were 2.33 higher than the odds in the multiparous women [AOR = 2.33, 95%CI (1.26–4.29)].

**Conclusions:**

This study revealed that the magnitude of failed induction was higher when compared to other similar findings. Bishop scores, membrane status, and parity were significantly associated factors with failed induction. Preparation of the cervix before commencing induction is recommended to improve induction success.

## Introduction

Induction of labor is a medical iatrogenic stimulation of uterine contraction before the spontaneous onset of labor to achieve vaginal delivery [1]. It is one of the most common procedures in obstetrics and one of the fastest-growing procedures in the world mainly in developed countries [2]. The common indications include membrane rupture without labor, gestational hypertension, oligohydramnios, non-reassuring fetal status, post-term pregnancy, and various maternal medical conditions such as chronic hypertension and diabetes [3, 4].

A workshop convened by the United States National Institute of Child Health and Human Development (NICHD), Society of Maternal-Fetal Medicine (SMFM), and American College of Obstetricians and Gynecologists (ACOG) proposed that failed induction be defined as the failure to generate regular contractions approximately every three minutes and cervical change after at least 24 hours of oxytocin administration [5]. There is no universal standard for what constitutes a failed induction, the key principle is to allow adequate time for cervical ripening and development of an active labor pattern before determining that induction has failed [6].

There is no single global figure that indicates the magnitude of failed induction of labor [7]. The magnitude of failed induction differs according to the induction guideline, what constitutes failed induction, and the induction method used [8, 9]. It was reported to be 18% in Nigeria [10], 7.2% in Mekelle [11], 17.3% in Hawassa referral hospital [12], 19.7% in Dessie Referral Hospital [13], 21.4% in Jimma University specialized hospital [14]. According to a study done in Kathmandu University Medical, Nepal the failure rate of induction can be higher than 50% depending on the indication for induction [4].

Parity, pre-labor rupture of membrane, pre-induction bishop score, and age of the mother were factors that were identified to be associated with failed induction of labor [7, 11, 13, 15–19]. Failed induction usually results in a cesarean section, which is a more potential health risk to the woman and the baby [3]. The cesarean section also results in a significantly longer recovery period, bleeding, possible injury to organs such as the bowel or bladder, adhesions leading to future pregnancy complications, and postoperative complications [3, 7, 20]. After a cesarean section, the neonate needs more breathing and immediate care than vaginal delivery [18]. Rarely, the baby might be nicked or cut during the cesarean incision [18].

Most previous studies regarding the magnitude of failed inductions and associated factors were conducted on primigravida and post-term pregnancies. Additionally, the previous studies conducted in the country mainly focused on secondary data (record review) which in most cases are incomplete with missing important information. Thus, this study included pregnancies from 28 and above weeks of gestation. The result of this study is used for policymakers and the health system. The result of this study is also used as a baseline for other researchers regarding the failed inductions. Therefore, this study was aimed to assess the magnitude and factors associated with failed induction of labor of pregnant women attending the labor ward of Adama hospital medical college (AHMC) for induction of labor.

## Methods

### Study area

The study was conducted at Adama Hospital Medical College (AHMC) which is located in Adama city. Adama city is located in the East Showa zone, Oromia regional state, and 99KM far from the capital city, Addis Ababa. The city has a total population of 250, 000 according to the 2007 Central Statistics agency (CSA) report. The city has one governmental and three private hospitals and five health centers. AHMC is one of the largest referral hospitals for nearby zones and regions (Afar, Amhara, and Somali regions) which give services to about five million people. The hospital has an operation room with 6 functional operation tables, eight gynecologists, 20 anesthetists, and 23 midwives. The average annual number of deliveries of all types in this hospital is 7800 and monthly about 650 women give birth in the hospital. On average about five women are admitted for induction of labor per day (AHMC).

### Study design and period

A hospital-based cross-sectional study was conducted from 1st of January to 30th April 2020.

### Source and study population

All pregnant women admitted to the labor ward of AHMC for induction of labor were source population and all pregnant women admitted to labor ward of AHMC for induction of labor during the study period and gestational age of 28 weeks and above from reliable last normal menstrual period (LNMP) or early ultrasound (U/S) (< 22 weeks), cephalic presentation who are admitted for induction of labor were study population. Women with any condition precluding vaginal delivery including, estimated fetal weight (EFW)>4500gm, previous uterine scar, malpresentation, antepartum hemorrhage (APH), (placenta previa), abnormal cervical anatomy, or cervical cerclage were excluded.

### Sample size determination and sampling procedures

The sample size was calculated by using Epi info version 7.2.4.0 software with the assumptions of 95% two-sided confidence level (CI), Power of 80% which is the conventional choice [21], and the study done in Hawassa Public Health Facilities, Ethiopia [17]. The maximum sample size amongst the variables was the final sample size for the study, which is 266, and adding a 10% non-respondent rate, the final sample size was 293.

The study units were allocated using a systematic random sampling technique. Study subjects were selected every second individual by dividing the previous year’s six-month report of pregnant women admitted to the labor ward for induction of labor (500) divided by the total sample.

### Measurements

#### Failed induction

Is declared when there has been no cervical change or descent of the presenting part after 6–8 hours of labor, or contractions of 3 in 10 minutes or 1 every 3 minutes has not been achieved [1].

#### Favorable bishop score

Score of cervical dilation, effacement (%), station, consistency, and position >5 shows induction is likely to succeed [1].

### Data collection tools, procedure, and quality control

Data were collected through interviewer-administered semi-structured questionnaires by using the Afan Oromo version questionnaire and data extraction checklists which were prepared reviewing different related literature conducted previously and then translated to Afan Oromo and back to English by language experts to check the consistency. The questionnaire contains socio-demographic characteristics, past medical and obstetric history, current obstetric, neonatal-related factors, and induction related characteristics. Data were collected through a face-to-face interview by using a semi-structured Afan Oromo version questionnaire and using a data extraction checklist for the different clinical observations from maternal records. Data were collected by five -year-two obstetrics and gynecology residents and one supervisor from year-three obstetrics and gynecology resident.

Data quality was assured during collection, coding, entry, and analysis. The two-day training was given for the data collectors and supervisors before actual data collection. The collected data were reviewed and checked for consistency, clarity; completeness, and accuracy throughout the data collection process by data collectors and a supervisor. Also, a reliability estimate of the selected data was conducted and the Cronbach’s Alpha of the reliability statistics were 0.681.

### Data processing & analysis

The collected data was entered into Epi-data Version 4.6 and exported to SPSS version 23 for analysis. Descriptive statistics like frequency tables, graphs, and descriptive summaries were used to describe the study variables. Both bivariate and multivariable logistic regressions were performed to identify significant factors associated with failed induction. Those variables with p<0.25 in the bivariate analysis were considered for multivariable logistic regression analysis. Since the outcome variable under consideration in this study is dichotomous with a ‘yes/no response, the Hosmer-Lemeshow goodness of fit test was used to check model fitness before running the final model with a p-value of 0.75 [22]. Finally, screened variables were fitted to the multivariable logistic regression model through a backward stepwise method to reduce the effects of cofounders and to identify the independent effects of each variable on the outcome variable. An adjusted odds ratio for a 95% confidence interval was employed for the strength and directions of association between independent variables and the outcome variables. A P-value of <0.05 was used to declare statistical significance.

### Ethics approval and consent to participate

Ethical clearance was obtained from the ethical review committee of Adama Hospital Medical College. Permission paper was obtained from the obstetrics and gynecology department. Data were collected after full informed written consent was obtained and confidentiality of the information was maintained by excluding names as identification in the questionnaire and keeping their privacy during the interview and examination. Besides, each respondent was assured that the information provided by them would be confidential and used only for research.

## Results

### Socio-demographic characteristics of the study participants

In this study, a total of 293 mothers with a gestational age of greater than 28 weeks and who indicated induction were included with a full response rate. One hundred fourteen (39.9%) of the participants were between the ages of 25–29 years and 258 (88.1%) of them were urban residents. Regarding occupation, 129(44%) of them were housewives, 192(65.5%) respondents were orthodox in religion and 121(47.3%) of them were Amhara in ethnicity. Two hundred eighty-four (96.9%) of the participants were married. Concerning the BMI of mothers, 167(57%) were overweight (Table 1).

**Table 1:** Distribution of socio-demographic characteristics of the study participants in Adama hospital medical college, 2020 (n = 293).

Characteristics	Frequency	Percentage
**Maternal Age**		
15–19 years	13	4.4
20–24 years	79	27
25–29 years	114	38.9
30–34 years	64	21.8
35–39 years	21	7.2
40 years and above	2	0.7
**Ethnicity**		
Oromo	102	34.8
Amhara	121	47.3
Tigre	28	9.6
Gurage	32	10.9
Others^1^	10	3.4
**Occupation**		
Housewife	129	44
Merchant	47	16
Government employee	62	21.2
Others^2^	55	18.8
**Place of residence**		
Urban	258	88.1
Rural	35	11.9
**Religion**		
Orthodox	192	65.5
Muslim	60	20.5
Protestant	38	13
Catholic	2	0.7
Others^3^	1	0.3
**Marital status**		
Married	284	96.9
Single	6	2
Divorced	3	1
**BMI**		
Underweight	3	1
Normal	68	23.2
Overweight	167	57
Obese	55	18.8

^1^: Afar, Wolaita, Somali, Hamar,

^2^: daily laborer, self-employed, unemployed,

^3^: wakefata.

### Obstetric and fetal characteristics

Almost half (50.2%) of the study participants were primigravida and a majority (94.5%) of them had ANC follow up and 178(64.3%) of them followed their ANC at a health center. Moreover, 154 (56.4%) mothers had followed their ANC three and above times. Regarding their gestational age, 161 (54.9%) of them had on the term. One hundred seventy-seven (60.4%) of study participants had intact membranes before initiation of induction and 228 (77.8%) babies had normal birth weight (Table 2).

**Table 2:** Obstetric and fetal characteristics of study participants, in Adama Hospital Medical College, East Shoa, Ethiopia, 2020 (n = 293).

Characteristics	Frequency	Percent
**Parity**		
Primigravida	147	50.2
Parous	146	49.8
**ANC**		
Yes	277	94.5
No	16	5.5
**Facility for ANC (n = 277)**		
Hospital	57	20.6
Health center	178	64.3
Private clinic	42	15.2
**Number of ANC visit(n = 277)**		
One	20	7.3
Two	99	36.3
Three and above	154	56.4
**Gestational age**		
Preterm	81	27.6
Term	161	54.9
Post-term	51	17.7
**Membrane status**		
Intact	177	60.4
Ruptured	116	39.6
**Birth weight**		
Very low birth weight	15	5.1
Low birth weight	46	15.1
Normal birth weight	228	77.8
Big baby	4	1.4
**Mode of delivery**		
Vaginal	175	59.7
Operative vaginal delivery	3	1
CD	115	39.2
**Birth outcome**		
Alive	275	93.9
Dead	18	6.1
**Sex of the newborn**		
Female	176	60.1
Male	117	39.1
**APGAR at the first minute**		
Normal	238	81.2
Intermediate	36	12.1
**APGAR at the fifth minute**		
Normal	268	91.5
Intermediate	7	2.4

### Ripening and induction related characteristics

**Fig 1:**
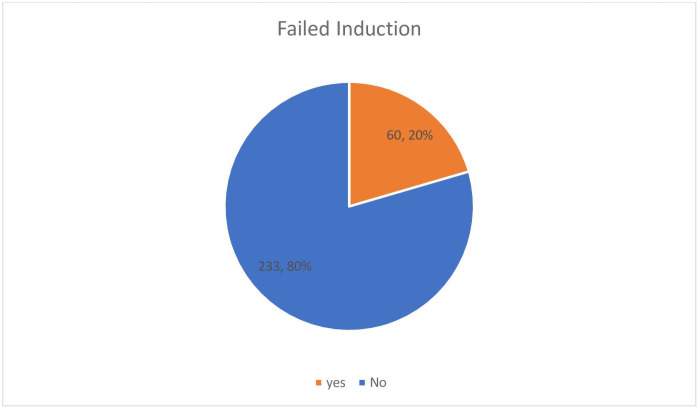
Prevalence of failed induction among study participants in Adama Hospital Medical College. East Shoa, Oromia, Ethiopia, 2020.

Among the 293 participants, PROM, PIH, post-term, and oligohydramnios were the indication for induction in 96 (32.8%), 65 (22.2%), 50 (17.1%), and 43 (14.7%) of the mothers respectively. Two hundred forty participants (81.9%) had unfavorable bishop scores at admission and of this for 139 (57.9%) mothers; a balloon catheter was used for ripening (Table 3). The magnitude of failed induction was 60 (20.5%) with a 95% CI of 15.7–25.3 (Fig 1).

**Table 3:** Ripening and induction-related characteristics of the study participants, in Adama Hospital Medical College, East Shoa, Ethiopia, 2020 (n = 293).

Characteristics	Frequency	Percentage
**Indication for induction**		
Post-term	50	17.1
PIH	65	22.2
DM	5	1.7
Oligohydramnios	43	14.7
PROM	96	32.8
Congenital anomaly	2	0.7
IUFD	16	5.5
APH (Abruption)	16	5.5
**Bishop scores**		
Favorable	53	18.1
Unfavorable	240	81.9
**Methods of ripening(n = 240)**		
Balloon catheter	139	57.9
Sublingual misoprostol	78	32.5
Vaginal misoprostol	23	9.6
**Methods of induction**		
Misoprostol alone	2	0.7
Oxytocin alone	291	99.3

### Factors associated with failed induction

Binary logistic regression was fitted to assess the association between failed induction and associated factors. Factors including, bishop scores, membrane status, and parity were significantly associated with failed induction in the bivariate logistic regression at a p-value of 0.25 (Table 4) and included in the final multivariable logistic analysis. In the final multivariable logistic regression; parity, the status of the membrane at the start of induction, and bishop score are significantly associated with failed induction at a p-value of 0.05.

**Table 4:** Bivariate logistic regression analysis showing factors associated with failed induction in Adama Hospital Medical College, East Shoa, Oromia, Ethiopia, 2020, (n = 293).

	Failed induction		COR [95% C.I.]	P-value
	Yes	No		
**Maternal age**				.036
< = 24 years	16	76	1.404 (.372–5.295)	.617
25–29 years	33	81	2.716 (.756–9.761)	.126
30–34 years	8	56	.952(.230–3.947)	.946
35 years and above	3	20	1	
**ANC follow-up**				
Yes	56	221	1	
No	4	12	1.315 .409 4.234	0.646
**History of medical disorder**				
Yes	6	13	1.898 (.689–5.227)	0.215
No	53	218	1	
**Bishop score**				
Favorable	3	50	1	
Unfavorable	57	183	5.19[1.56–17.28]	0.007
**Parity**				
Primigravida	40	107	2.36[1.30–4.27]	0.005
Multipara	20	126	1	
**Membrane status**				
Intact	45	32	2.30[1.21–4.35.]	0.011
Ruptured	15	101	1	

The odds of failed induction in unfavorable bishop score were 4.05 higher than the odds in favorable bishop [AOR = 4.05 95%CI (1.19–13.77)]. The odds of failed induction in primigravida were 2.33 higher than in the multiparous women [AOR = 2.33, 95% CI (1.26–4.29)]. The odds of failed induction in an intact membrane were 2.05 higher than the odds in the ruptured membrane. [AOR = 2.05, 95%CI (1.06–3.98)] (Table 5).

**Table 5:** Multivariable logistic regression analysis showing factors associated with failed induction in Adama Hospital Medical College, East Shoa, Oromia, Ethiopia, 2020, (n = 293).

	Failed induction		AOR [95% C.I.]	P-value
	Yes	No		
**Bishop score**				
Favorable	3	50	1	
Unfavorable	57	183	4.05[1.19–13.77]	0.025
**Parity**				
Primigravida	40	107	2.33[1.26–4.29]	0.007
Multipara	20	126	1	
**Membrane status**				
Intact	45	32	2.05[1.06–3.98]	0.033
Ruptured	15	101	1	

## Discussions

Induction of labor is one of the commonly performed obstetric procedures nowadays. This increment in labor induction especially if not indicated is a concern as it leads to unnecessary cesarean section.

The prevalence of failed induction according to the current study was 20.5% with a 95% CI of (15.7–25.3). This finding is comparable with the study done in Jimma University specialized hospital, 21.4% [14]. The current finding is also in line with a study done in Hawassa public health facilities, 17.3% [17]. The result of the current study is also comparable with the study done in Dessie referral hospital (19.7%) [13]. This is necessarily due to the similarity in the definition of failed induction time which is six to eight hours in the national induction protocol developed by the ministry of health [1].

The prevalence of failed induction in this study is in line with the study in Aga Khan University Hospital; Karachi in Pakistan (18%) [18]. This might be due to failure in maintaining the serum oxytocin concentration during a change of infusion bag and additionally the 20 minutes dose increment is not enough to achieve vaginal delivery as 40 minutes is needed to achieve a steady oxytocin serum level [23]. Another possible reason for the high rate of failure could be the limited time that is used to define failed induction (6 to 8 hours) in the study area [1] in contrary to the study conducted in Karachi which defined failure of induction based on the mode of delivery [18]. Another research done in India reported that the prevalence of failed induction was 50.5% [24]. This difference might be due to the failure of induction in the latter study was considered based on the mode of delivery.

According to this study, the odds of failed induction in unfavorable Bishop score was 4.05 higher than the odds in favorable Bishop score. The current finding is in line with the study done in Hawassa public facilities [17] and Jimma University Specialized Hospital [14]. Similarly, this finding is in agreement with studies done in Amhara region referral hospitals [4] and Dessie referral hospitals [20]. The possible explanation might be because cervical repining involves the enzymatic dissolution of collagen fibrils, increase in water content, and chemical changes [7].

The current finding is also supported by the study done in the Aga Khan University Hospital, Karachi in Pakistan [18] Similarly, the study done in Ankara Maternity and Women’s Health Teaching Hospital, Turkey on unsuccessful labor induction in women with unfavorable cervical scores showed that unfavorable Bishop score and failed induction were significantly associated [25].

According to this study, the odds of failed induction in primigravida were 2.33 higher than the odds in the multiparous women. This finding is in line with studies done in Hawassa public facilities [17], Jimma University specialized hospital [14], Amhara region referral hospitals [19], and Dessie referral hospital [13]. Another study was done in Aga Khan University Hospital; Karachi in Pakistan supported the present finding [18]. This might be due to the nature of the cervix in multiparous women which is a wide, uneven, and bulky appearance that makes it easy to dilate[3, 7].

This study also showed that the odds of failed induction in the intact membrane were 2.05 higher than the odds in the ruptured membrane. This finding is in line with the study done in Hawassa public health facilities [17]. Another study was done in Ankara Maternity and Women’s Health Teaching Hospital; Turkey is also consistent with the current finding [25]. This has to do with the contents of amniotic fluid, prostaglandins, which are responsible for cervical dilatation and hence successful birth [3, 7].

However, the study done in Jimma University specialized hospital is not in agreement with the current finding [14]. This difference could be due to the fact the current study used both face-to-face interviews and data extraction methods while the other studies mostly employed secondary data which could expose it to incomplete and missing important data. Another explanation might be due to some practices like delayed amniotomy in the active stage of labor due to fear of cord prolapse if amniotomy is conducted in early labor [17].

### Limitations of the study

The study could be prone to recall bias since data were from the mothers’ backward history. It is also difficult to establish a cause-and-effect relationship because of the cross-sectional nature of the study.

## Conclusions

This study revealed that the magnitude of failed induction was higher when compared to other similar findings. Unfavorable bishop scores, intact membrane status, and primigravida were significantly associated factors with failed induction.

## Supporting information

S1 File (PDF)

S1 Data (SAV)

## Abbreviations

AHMC: Adama Hospital Medical College
ANC: Antenatal Care
PIH: Pregnancy induced hypertension
PROM: premature rupture of membranes
