# Supplementary material for: The magnitude of failed induction and associated factors among women admitted to Adama hospital medical college: A cross-sectional study
Source: PLoS One. 2022 Jan 27;17(1):e0262256. doi: 10.1371/journal.pone.0262256 (PMC8794164; doi:10.1371/journal.pone.0262256)
Supplement: S1 File — (PDF) [file pone.0262256.s001.pdf]

1. English Version Questionnaire

1. **Consent form that certifies the respondent's agreement**

Hello. My name is **---------- and** we are working a research on magnitude of failed induction and associated factors among pregnant women admitted to labor ward of AHMC for induction of labor.

The objective of this study was to assess magnitude of failed induction and associated factors among pregnant women admitted to labor ward of AHMC for induction of labor. This information will help the hospital to prepare management guideline for pregnant women who were admitted for similar procedure. Therefore, your honest and genuine participation by responding to the interview and allowing to your chart is highly appreciated and helpful to attain the objective of the study.

Confidentiality: Whatever information you provide will be kept strictly confidential, and will not be shared with anyone other than members of our study team.

Withdrawal without prejudice: Participation in this study is voluntary, and if you don't want to answer any question, inform me and I will go to the next question; or you can stop the interview at any time. However, we hope you will participate in the study since your views are important.

Risk and benefits: There are no risks or discomforts from your participation in the study.

The benefits of anticipation are the opportunity to treat problems and to develop management guideline. There is no payment for participation in this study.

Agreement: At this time, do you want to ask me anything about the study?

May I begin the interview now? 1. yes---- 2. No----

If your answer is yes, you can start answering the questions. But if your answer is no, thank you, your will stop here.

Signature of respondent: --------------- Signature of data collector--------------- date__________

Thank you

Identification:

1. Questionnaire code_________________

2. Date of admission _______________

3. Name of data collector______________________

Interview based questionnaires for part I and Part II:

**Part I: Socio-demographic variables**			
**S. N**	**Questions**	**Answers**	**Code**
**1.**	Age?		/<br>_____/
**2.**	Weight		
**3.**	Height		
**4.**	Ethnicity	1. Oromo<br>2. Amhara<br>3. Tigre<br>4. Gurage<br>5. Others specify	
**5.**	Occupation?	1. Housewife,<br>2. Merchant,<br>3. government employee,<br>4. other	/<br>_____/
**6.**	Residence	1. Urban<br>2. Rural	/<br>______/
**7.**	Religion	1. Orthodox<br>2. Muslim<br>3. protestant<br>4. catholic<br>5. other	/<br>_____/
**8.**	What is your marital status?	1. Married<br>2. Single<br>3. Divorced	

		4. widowed,<br>5. other (specify)	/<br>_____/
**9.**	What is your monthly income in birr?	_______	/_____/
**Part II: Obstetric and Medical history**			
**1.**	Parity?	Primigravida ____<br>Parous	/<br>_____/<br>/<br>_____/
**2.**	Did you have ANC follow up for the current pregnancy?	1. yes<br>2. No	/<br>_____/
**3.**	If yes, for the above question, to which facility?	1. Hospital<br>2. Healthy center<br>3. private clinic	/<br>_____/
**4.**	If yes for ANC follow up, how many visits you attended?	1. one<br>2. two<br>3. three and above	/<br>_____/
**5.**	Do you have any history of medical disorders?	1. yes<br>2. no	/<br>_____/
**6.**	For the above question, if yes, what?	1. Hypertension<br>2. Diabetes mellitus<br>3. other (specify)	/<br>_____/
**7.**	During current pregnancy, did you inform any problem	1. Yes<br>2. No	/<br>_____/
**8.**	If yes for the above question, what?	1. Hypertension<br>2. Diabetes mellitus<br>3. Fetal congenital anomaly	/<br>_____/

		4. Other (specify)	
**9.**	Date and time of initiation of induction		
**10.**	Date and time of delivery		
**11.**	Membrane status	Intact________ruptured ______	

Part III: Feto-Maternal assessment upon admission:

1. Gestational age: from reliable LNMP___ weeks or from early U/S_____ weeks,
2. FHB. a. positive___ b. negative____
3. Obstetric U/S:

   3.1 Gestational age___ weeks.

   3.2 EFW________grams

   3.3 Gross congenital anomaly? Yes____ No____
4. Based on the U/S findings, is the fetus macrosomic? A. Yes___ b. No___
5. Biophysical profile results__ a. >=6 b. ≤ 4
6. At the time of admission, what is the bishops score? A. ≤5 ________ B> 5_________

7. Indications for induction

A. post term B. PIH C. DM D. Oligohydramnios E. PROM F. congenital anomaly G. IUFD H. APH/abruption.

8. Methods of cervical ripening?

a) Balloon catheter ______
b) Sublingual misoprostol ______
c) Vaginal misoprostol _____

9. Methods of induction:

a. Misoprostol alone _______
b. Oxytocin infusion alone_______

Part IV: Obstetric outcomes:

1. Any instrumental delivery?   a. yes____   b. No______
2. If yes, why?
   a. Prolonged SSOL_______
   b. NRFHRP_______
   c. Meconium   stained amniotic fluid_________
   d. Shortening of SSOL_________
   e. Other (specify)__________
3. Any complications during induction of labor? (more than one option is possible to be selected)
   a. NRFHRP__
   b. MSAF__
   c. uterine rupture_________
   d. hyperstimulation_______

4. Mode of delivery

   a. SVD_____
   b. Operative vaginal delivery____
   c. Cesarean section____

5. If cesarean section, indication?

   a. Failed induction_____
   b. MSAF __
   c. NRFHRP ______
   d. Others_____

Part V: Neonatal outcomes:

1. Birth status: a. Alive___   b. Dead ___
2. Sex: a. male_____ b. female____
3. Weight (gm): _______
4. APGAR score: a. 1st minute ___ b. 5th minute ____

2. Afaan Oromoo version Questionnaire

Unka walii galtee qorannoo kana keessatti hirmaachuu fi hirmaachuu dhabuu:

Kabajamtoota hirmaattota:

Akkam jirtu? Ani **----------------**jedhama. Kanan hojjedhu Koollejjii Fayyaa Hospitaala Adaamaa, kutaa ulfaa fi Gadammeessaa keessa.

Kaayyoon qorannoo kanaa sababoota ciniinsuu qorichaan jalqabamuu galma gahuu dadhabuu waliin wal qabata. Bu'aan qorannoo kanaa immoo, hospitaalichi rakkoowwan walfakkaataa tahan irratti wal'aansaaf kan fayyadu qajeelfama qopheessuuf fayyada. Kanaafuu, hirmaannaan isin gaaffii fi deebii kanaaf taasistan fi kaardii keessan irraa ragaalee barbaachisoo tahan fudhachuuf taasistan galma ga'insa qorannoo kanaatiif baay'ee barbaachisaadha.

Icciitii eeguu: Odeeffannoon isin naaf kennitan iccitiin isaa ni eeggama akkasumas nama biraaf dabarsamee hin kennamu, warroottan qorannoo kana irratti hirmaataniif malee.

Haalduree tokko malee addaan kutuu: Hirmaannaan qorannoo kana keessatti taasistu fedhii irratti kan hundaa'edha. Yeroo barbaaddetti keessaa bahuu fi addaan kutuu ni dandeessa. Yoo gaaffiin isin deebisuu hin barbaanne jiraate natti himaa, gara gaaffii itti aanuttin darba.

Bu'aa fi miidhaa qorannichaa: Qorannoo kana keessatti hirmaachuun miidhaa takkalee hin qabu. Yookaan dhukkubbii hin uumu. Bu'aan qorannoo kana keessatti hirmaachuu, carruma kanaan wal'aansa argachuu fi qajeelfama rakkoo akkanaa wal'aanuuf fayyadu qopheessuuf.

Qorannoo kana keessatti hirmaachuu keessaniif kafaltiin isiniif kennamu hin jiraatu.

Walii galtee: Wanti ifa siif hin ta'iin yoo jiraate gafaachuuu ni dandeessu.

Kanaafuu, gaaffii koo eegaluu ni danda'aa? a. Eeyyeen_____ b. Lakki____

Yoo deebiin keessan eeyyeen tahe, gaaffii koo deebisuu ni dandeessu. Garuu, yoo deebii keessan miti tahe, galaatoomaa.

Mallattoo gaaffii deebisaa_______ Mallattoo qorataa___________ Guyyaa_______

Kutaa 1: Haala hawaas-dinagdee haadholii

**S. N**	**Gaaffilee**	**Deebii**	**K oodii**
**1.**	Umrii?		/<br>_____/
**2.**	Ulfaatina		
**3.**	Dheerina		
**4.**	Sanyii (ethnicity)	1. Oromoo<br>2. Amaaraa<br>3. Tigree<br>4. Guraagee<br>5. Kan biroo	
**5.**	Hojii Haadhaa?	1. Haadha manaa<br>2. daldaltuu<br>3. Hojjettuu mootummaa<br>4. kan biroo	/<br>_____/
**6.**	Bakka jireenyaa	1. magaala<br>2. baadiyyaa	/<br>______/
**7.**	Amantii	1. Ortoodoksii<br>2. Musliima<br>3. protestaantii<br>4. kaatolikii<br>5. kan biroo	/<br>_____/
**8.**	Haalli gaa'ela kee akkami?	1. heerumeera<br>2. hin heerumne<br>3. adda baaneerra<br>4. narraa du'eera<br>5. kan biroo	/<br>_____/

**9.**	Ji’aan tilmaamaan qarshii hammam argatta?	_______	**/_____/**

**Kutaa 2: Gaaffilee walhormaata waliin walqabatan**			
**1.**	Yeroo meeqa deesse (Parity)?	Primigravida ____<br>Parous	**/_____/**<br>**/_____/**
**2.**	Yeroo ulfa isa kanaa hordoffii gooteettaa?	1. eyyee<br>2.lakki	**/_____/**
**3.**	Eyyee yoo ta’e, dhaabbata fayyaa kamitti goote?	1. Hospitaala<br>2. Buufatafayyaa<br>3. kilinikadhuunfaa	**/_____/**
**4.**	Hordoffii gootee jirta yoo tahe, yeroo meeqaaf hordoffii goote?	--------	**/_____/**
**5.**	Dhukkuba beekame duraan qabdu jiraa? (Medical disorders)	1. eyyee<br>2. lakki	**/_____/**
**6.**	Yoo qabaatte maal ture?	1. dhiibbaa dhiigaa<br>2. Dhibee sukkaaraa<br>3. kan biro	**/_____/**
**7.**	Yeroo ulfa kanaa rakkoo simudate jiraa?	1. eyyee<br>2. lakki	**/_____/**
**8.**	Gaaffii oliif eyyee yoo jette maal ture?	1. dhiibbaa dhiigaa<br>2. Dhibee sukkaaraa<br>3. Fetal congenital anomaly	**/_____/**

		4. kanbiroo (ibsi)	
**9.**	Sa’aatii fi guyyaa “induction” jalqabame		
**10.**	Guyyaa fi sa’aatii itti deesse		
**11.**	Membrane status	Intact________ruptured ______	
